# Sex and Gendered Approach in Chronic Coronary Disease Guidelines: One Size Does Not Fit All

**DOI:** 10.1016/j.jacadv.2024.100859

**Published:** 2024-03-06

**Authors:** Cameron Blazoski, Yoo Jin Kim, Jared Spitz, Roger S. Blumenthal, Garima Sharma

**Affiliations:** a Department of Medicine, Johns Hopkins University School of Medicine, Baltimore, Maryland, USA; b Inova Schar Heart and Vascular, Inova Health System, Falls Church, Virginia, USA; c Ciccarone Center for the Prevention of Cardiovascular Disease, Johns Hopkins University School of Medicine, Baltimore, Maryland, USA

**Keywords:** lactation, pregnancy, sex and gender, spontaneous coronary artery disease

Chronic coronary disease (CCD) is a broad clinical diagnosis that groups obstructive and nonobstructive coronary artery disease (CAD).^1^ In 2022, an estimated 20.1 million persons living in the United States were classified as having CCD, and coronary heart disease remains the leading cause of death in the United States and worldwide.^2^ Over the last decade, there have been significant changes and practice developments in the management of CCD, especially pertaining to individuals identifying as women with CCD. The intertwining of sex-based differences in genetic and hormonal mechanisms with the complex dimension of gender and its different components and determinants that result in different disease phenotypes in women and men has been elucidated in recent years. Our understanding in the relative contribution of biological factors as it relates to cardiovascular phenotypes is still evolving. The 2023 CCD Guideline serves as an update to the 2012 Guideline and the 2014 Focused Update. The Guideline provides clinicians with a patient-centered, evidence-based approach to managing patients with CCD. This addition to the guidelines in the management of chronic coronary artery disease provides a nuanced and balanced perspective focusing on the pathophysiology and management of ischemic heart disease in both sexes. For the purpose of this article, we use sex and gender interchangeably recognizing the limitations in that approach.

## The need for a sex and gendered approach

Recent research and practices regarding treating pregnant, lactating, menopausal, and transgender individuals have required updates to guideline recommendations. Additionally, newer imaging and catheterization-driven phenotypes of microvascular angina have led to the streamlining of care pathways for chest pain in female patients. The 2023 CCD Guideline outlines the optimal management of patients with CCD and includes new sex- and gender-based recommendations. It provides an important update on the management of patients with CCD and specifically addresses critical time windows, from pregnancy to menopause. Given the sex- and gender-based disparities in the management of cardiovascular disease, these recommendations address a critical care gap.

The 2012 Guideline and 2014 Focused Update notably lacked evidence-based recommendations on managing women with CCD, but the 2023 Guideline provides new and updated recommendations and discussions on managing pregnant, lactating, postmenopausal, and transgender individuals with CCD and related cardiovascular diseases. These guidelines address critical time and transition windows. This paper summarizes changes in recommendations and addresses patient-centered ways to optimize clinical practice. Table 1 broadly compares and contrasts prior and current sex- and gender-specific recommendations. Of note, this article defines women as those who self-identify as women.

**Table 1:** Comparison of the 2012 and 2023 CCD Guideline’s Recommendations on Women Patients

Topic	2012 Guideline		2023 Guideline		Changes in 2023 Guideline
	Text	Official Recommendations	Text	Official Recommendations	
Pregnancy and lactation	N/A	N/A	Use of risk stratification scores in pregnancy, including a Table of CARPREG-II risk scoring system. Pharmacotherapy in Pregnancy and Lactation: safety of CV medications in pregnancy. Team-based approach to cardio-obstetrics care formally recognized.	Individuals with CCD contemplating pregnancy or who are pregnant should receive care from a multidisciplinary cardio-obstetric care team beginning before conception and continuing throughout postpartum state to improve maternal and fetal outcomes (Class 1, C-LD). Those contemplating pregnancy or who are pregnant should be risk-stratified and counseled regarding risks of adverse maternal, obstetric, and fetal outcomes (Class 1, C-LD). Medications: Continuation of statin use during pregnancy may be considered (Class 2, C-LD). Contraindication for RAAS inhibitors during pregnancy to prevent harm to the fetus (Class 3: Harm, C-LD).	New (and first) recommendations on managing pregnant and lactating patients with supporting evidence.
Menopause	Discusses risks of HT but it may be continued if prescribed for non-CV indications.	ET is not recommended in postmenopausal women with SIHD with intent of reducing CV risk or improving outcomes. 811-814 (Class 3: Harm, A).	The guideline discusses risks of HT and the lack of benefits for patients with CCD.	No postmenopausal HT because of a lack of benefit on MACE and mortality, and an increased risk of thromboembolism (Class 3: Harm, A).	Change in recommendation that patients with CCD should not receive postmenopausal HT.
Transgender individuals	N/A	N/A	Transgender individuals have increased rates of venous thromboembolism, acute MI, and stroke compared with ciswomen, possibly due to hormone therapy.	N/A	New discussion of transgender individuals and their CV risk.
Spontaneous coronary artery dissection	N/A	N/A	Screening questions (medical history, family history, review of systems) for SCAD-associated arteriopathies and CT disorders (eg, fibromuscular dysplasia, vascular Ehlers-Danlos, Marfan syndrome, Loeys-Dietz syndrome).	In patients with CCD who have experienced SCAD, counseling should be provided regarding potential triggers and risk of SCAD recurrence (Class 1, C-LD). In SCAD, beta-blocker therapy may be reasonable (Class 2b, C-LD). Evaluating for underlying vasculopathies is reasonable to identify abnormalities in other vascular beds (Class 2a, C-LD).	First recommendations on managing patients with CCD and SCAD. New table to screen for SCAD-associated arteriopathies and CT disorders
Microvascular disease	No section specifically on MVD.	N/A	Focus on clinical criteria for suspecting microvascular angina 1. Symptoms of ischemia (angina, dyspnea) 2. Absence of obstructive CAD on anatomic imaging 3. Objective evidence of ischemia 4. Evidence of impaired microvascular function Diagnostic criteria for vasospastic angina: 1. Nitrate responsive angina 2. Transient ischemic ECG changes 3. Visualization of coronary artery spasm Invasive hemodynamic definitions of INOCA endotypes with pharmacotherapeutic recommendations based on each endotype. Recommendation-specific supporting research.	In symptomatic patients with nonobstructive CAD, a strategy of stratified medical therapy guided by invasive coronary physiologic testing can be useful for improving angina severity and QOL (Class 2a, B-R).	New (and first) recommendations on managing MVD.

CAD = coronary artery disease; CARPREG = Cardiac Disease in Pregnancy; CCD = chronic coronary disease; CT = connective tissue; CV = cardiovascular; ECG = electrocardiogram; ET = estrogen therapy; HT = hormone therapy; INOCA = ischemia with nonobstructive coronary arteries; MACE = major adverse cardiac events; MI = myocardial infarction; MVD = microvascular disease; QOL = quality of life; RAAS = renin-angiotensin-aldosterone system; SCAD = spontaneous coronary artery dissection; SIHD = stable ischemic heart disease.

## Inclusion of pregnancy windows and menopause and important transitions across the lifespan for female patients

CCD is found in <4% of all pregnant patients in published registries, but these patients are at a significantly higher risk of cardiovascular complications and adverse pregnancy outcomes.^1^ Thus, careful evaluation, monitoring, and management by a skilled, multidisciplinary team is crucial to address medication optimization, cardiovascular surveillance, and obstetric planning.^1^ Pregnancy outcomes generally improve with multidisciplinary care teams, especially involving a cardiologist trained in cardio-obstetrics.^1^ However, only about 29% of practicing cardiologists have received cardio-obstetrics training, and 76% of cardiology practices do not have a dedicated cardio-obstetrics care team.^3^

Women with CCD who are considering pregnancy require appropriate risk stratification. The CARPREG (Cardiac Disease in Pregnancy) II risk scoring system is a widely used system for estimating adverse cardiac events during pregnancy. It is a weighted score incorporating information about left ventricular function, valvular function, pulmonary hypertension, coronary disease, and aortic disease.^1^ The new guidelines provide a systemic approach to the management of established heart disease in pregnant patients. This is a significant change from the prior 2012 guidelines.

## Medications in pregnancy and lactation

Statin therapy has been contraindicated in pregnancy since their approval in 1987 due to concerns of potential teratogenicity and the role of cholesterol in the developing embryo, but recent studies have found that the increased risk of fetal malformations and miscarriage associated with statins is accounted for by underlying maternal comorbidities such as preexisting diabetes.^1^ However, because the benefit of statins may prevent potentially serious events in a small number of very high-risk women, the contraindication of statins in these women may not always be appropriate. Recently, the U.S. Food and Drug Administration requested the removal of the “Pregnancy Category X” label for statins and encouraged shared decision-making between physicians and patients.^1^

The decision regarding lipid-lowering therapy during pregnancy and lactation is one that must be made on an individual basis, after taking into account baseline atherosclerotic cardiovascular disease risk, lipid control, and the initial indication for lipid-lowering therapy. Two groups most likely to benefit from continuation of lipid-lowering therapy are those with a history of clinical atherosclerotic cardiovascular disease and those with homozygous familial hypercholesterolemia. Prior to conception, individuals on lipid-lowering therapy should have their risk factors optimized and lifestyle changes implemented. In general, statins should be stopped at least 1 month (preferably 3) prior to conception and not restarted until completion of breastfeeding. Additionally, there is no evidence to support the safety of ezetimibe, bempedoic acid, inclisiran, or evinacumab and therefore these drugs should be discontinued. In these limited cases where continuation of lipid-lowering therapy is considered, (re)initiation of therapy should be considered carefully only after the first trimester. Additionally, in these higher risk groups, consideration of lipoprotein apheresis is warranted.

## Menopause transition

An important highlight of the 2023 Guideline is the recognition of menopause transition and its impact on the cardiometabolic health of the individual. Previous observational studies indicated a possible cardiovascular benefit of postmenopausal hormone therapy, yielding the 2012 Guideline recommendation which approved estrogen therapy based on shared decision-making, even in patients with CCD. Recent trials have shown that hormone therapy has no benefit on major adverse cardiovascular events with a significantly increased risk of venous thromboembolism, resulting in a guideline recommendation that women with CCD should not receive hormone therapy.^1^ There remains significant discussion on the risk profile of systemic estrogen therapy, with some researchers indicating that estrogen therapy is low risk, if provided within 10 years of menopause. This new guidance lends a framework for clinicians for the management of vasomotor symptoms in the menopause transition.

## Inclusion of transgender patients

New to the CCD Guideline are the recommendations on transgender health. Transgender women have increased rates of venous thromboembolism, acute myocardial infarction (MI), and stroke compared with cisgender women but not always when compared with cisgender men.^1^ This is thought to be partially related to hormone therapy, but the 2023 Guideline emphasizes that future research is needed to appropriately assess the cardiovascular risk of hormone-treated transgender women with CCD.^1^

## Spontaneous coronary artery dissection management

Spontaneous coronary artery dissection (SCAD) is an under-recognized cause of MI that is more prevalent in women.^1^ Acute events are typically medically managed, although certain unstable patients may require revascularization. Following acute management of SCAD, the primary focus is preventing recurrences. While patients with SCAD are generally well-managed with conservative medical management, estimated 5-year recurrence rates can be up to 27%.^1^ The most common triggers for SCAD, either initial or recurrent, are emotional stress and physical exertion.^1^ Other observed triggers potentially include perimenopausal state, use of oral contraceptives, postmenopausal hormone therapy, infertility treatments, and high-dose corticosteroid administration.^1^ It is important for clinicians to investigate potential triggers and to educate on avoidance, if possible. There is little evidence on pharmacologic therapies specifically to prevent SCAD recurrence, but emerging data emphasize the potential benefit of beta-blocker therapy in the management of patients who have had SCAD.^1^

## Ischemia with nonobstructive coronary arteries

Ischemia with nonobstructive coronary arteries (INOCA), a form of myocardial ischemia caused by coronary vasomotor dysfunction, is present in >50% of patients who undergo invasive angiography due to angina. Patients can be classified as having chronic microvascular angina, vasospastic angina, or a mixed endotype. Patients with INOCA have anginal symptoms similar to those with obstructive CAD.^1^ Notably, women are predominantly affected by INOCA, but they are consistently under-recognized and undertreated. Among women with cardiac ischemia, 81% were reported to not have minimal to no CAD.^4^ Providers should work to identify INOCA, given its strong association with all-cause death and MI, especially in female patients.^1^

## A new sex and gendered approach to streamline heart disease management

The increased focus on sex- and gender-specific considerations in chronic heart disease in patients attempts to address the under-recognition of women with CCD.

Despite 44% of American women living with some form of heart disease,^2^ only 56% know that heart disease is the leading cause of death in U.S. women and only 37% were aware that it was the primary cause of maternal mortality.^5^ Awareness among women has increased slightly since initial surveys in 1997, but it has not improved significantly since 2006, especially in younger women and minority women, despite focused public education campaigns.^5^

Clinicians similarly under-recognize and, subsequently undertreated, cardiovascular disease in women. Physicians are more likely to underestimate the probability of cardiovascular disease in women and are more likely to assign a lower cardiovascular disease risk score to female patients when compared to risk-matched male patients.^6^ Female patients are also less likely to receive guideline-recommended statin intensity than male patients.^7^

Women are consistently under-represented in cardiovascular research studies. Of the 740 cardiovascular trials registered in ClinicalTrials.gov from 2010 to 2017, only 38.2% of trial participants were women.^8^ This under-representation is exacerbated in minority women. Given that women have different pathophysiology and manifestations of cardiovascular disease than men and are more likely to have their cardiovascular health impacted by socioeconomic, political, and cultural factors, a lack of research directly impacts the awareness and management of cardiovascular disease in women.^9^

A lack of research is a direct result of the disproportionate funding of research by sex or gender. A 2021 study found that the National Institutes of Health directs a disproportionate share of its resources to diseases that affect primarily men, at the expense of those that affect primarily women.^10^

## Conclusions

The 2023 CCD Guideline significantly addresses the under-recognition and undertreatment of women with cardiovascular disease by including specific recommendations pertaining to women’s cardiovascular and cardio-obstetric health, including additional focus on diseases like SCAD and INOCA that disproportionately impact women. Clinicians should also remain informed on the latest guidelines on medication use in pregnancy and ensure women are on appropriate and safe medications throughout the lifecycle and with pregnancy. Hopefully, increasing inclusion of sex and gendered approach in guidelines such as the 2023 CCD Guideline yields an increased focus on women’s cardiovascular health and, eventually, improved cardiovascular outcomes. These guidelines can provide a more streamline care pathway for the management of ischemic heart disease with the influence of biological and sociocultural differences between women and men, and taking into account the difference in cardiovascular disease outcomes, This “one size does not fit all” approach will lead to optimized treatments and improved health care for all patients.

## Funding support and author disclosures

Dr Sharma is supported by 10.13039/100000968AHA HRSN 979462. All other authors have reported that they have no relationships relevant to the contents of this paper to disclose.
